# Oral administration of bovine lactoferrin suppresses the progression of rheumatoid arthritis in an SKG mouse model

**DOI:** 10.1371/journal.pone.0263254

**Published:** 2022-02-11

**Authors:** Shunryou Yanagisawa, Karin Nagasaki, Chanbora Chea, Toshinori Ando, Nurina Febriyanti Ayuningtyas, Toshihiro Inubushi, Atsushi Ishikado, Hiromichi Imanaka, Eiji Sugiyama, Ichiro Takahashi, Mutsumi Miyauchi, Takashi Takata

**Affiliations:** 1 Department of Oral & Maxillofacial Pathobiology, Graduate School of Biomedical & Health Sciences, Hiroshima University, Hiroshima, Japan; 2 Department of Periodontology and Endodontology, Tohoku University Graduate School of Dentistry, Sendai, Japan; 3 Faculty of Dentistry, University of Puthisastra, Phnom Penh, Cambodia; 4 Center of Oral Clinical Examination, Hiroshima University Hospital, Hiroshima, Japan; 5 Faculty of Dental Medicine, Airlangga University, Surabaya, Indonesia; 6 Department of Orthodontics and Dentofacial Orthopedics, Graduate School of Dentistry, Osaka University, Osaka, Japan; 7 R&D Department, Sunstar Inc., Osaka, Japan; 8 Hiroshima University, Hiroshima, Japan; 9 Department of Mucosal Immunology, Graduate School of Biomedical & Health Sciences, Hiroshima University, Hiroshima, Japan; 10 Tokuyama University, Shunan, Japan; Nippon Medical School, JAPAN

## Abstract

Rheumatoid arthritis (RA) is an autoimmune disease characterized by inflammatory bone destruction in which tumor necrosis factor alpha (TNF-α) plays a key role. Bovine lactoferrin (bLF) is a multifunctional protein with anti-inflammatory and immunomodulatory properties. This study aimed to clarify the inhibitory effects of bLF on the pathological progression of RA. The mannan-induced arthritis model in SKG mice (genetic RA model) was used. Orally applied liposomal bLF (LbLF) markedly reduced ankle joint swelling and bone destruction. Histologically, pannus formation and osteoclastic bone destruction were prevented in the LbLF-treated animals. Moreover, orally administered LbLF improved the balance between Th17 cells and regulatory T cells isolated from the spleen of mannan-treated SKG mice. In an *in vitro* study, the anti-inflammatory effects of bLF on TNF-α-induced TNF-α production and downstream signaling pathways were analyzed in human synovial fibroblasts from RA patients (RASFs). bLF suppressed TNF-α production from RASFs by inhibiting the nuclear factor kappa B and mitogen-activated protein kinase pathways. The intracellular accumulation of bLF in RASFs increased in an applied bLF dose-dependent manner. Knockdown of the lipoprotein receptor-related protein-1 (LRP1) siRNA gene reduced bLF expression in RASFs, indicating that exogenously applied bLF was mainly internalized through LRP-1. Immunoprecipitated proteins with anti-TNF receptor-associated factor 2 (TRAF2; an adapter protein/ubiquitin ligase) included bLF, indicating that bLF binds directly to the TRAF2-TRADD-RIP complex. This indicates that LbLF may effectively prevent the pathological progression of RA by suppressing TNF-α production by binding to the TRAF2-TRADD-RIP complex from the RASFs in the pannus. Therefore, supplemental administration of LbLF may have a beneficial effect on preventive/therapeutic reagents for RA.

## Introduction

Rheumatoid arthritis (RA) is an autoimmune disease characterized by continuous inflammation and proliferation of synovial fibroblasts, resulting in irreversible destruction of articular cartilage and bone [1]. The worldwide prevalence of RA varies between 0.5% and 1%, with RA being more common in women [2]. RA begins with joint stiffness, followed by swelling and pain in the affected joint. Eventually, RA leads to body dysfunction owing to marked deformity of the joint [3]. Therefore, the quality of life of patients significantly decreases with RA progression. Although it is well accepted that genetic, immunological, and environmental factors contribute to the onset of RA, the cause and detailed pathogenic mechanism of RA are not still well clarified.

The pathological characteristics of RA include pannus formation and destruction of the affected joint. Pannus mainly consists of aggressive synovial fibroblasts with inflammatory cell infiltration, such as macrophages and lymphocytes [4]. Proinflammatory cytokines produced from the pannus play an important role in the pathological progression of RA. Tumor necrosis factor alpha (TNF-α), which has multiple functions, including the induction of other inflammatory cytokines [5], proliferation and activation of synovial fibroblasts [6], and induction of osteoclastic bone resorption [7], is a key cytokine in RA pathogenesis and progression.

Lactoferrin (LF), an 80 kD iron binding glycoprotein in the transferrin family [8], is known to play a critical role in defense against infection [9]. Moreover, LF has multi-functional effects, including anti-inflammatory [10], immunomodulatory [11], and antitumor effects [12]. As LF is the second largest component of milk protein after casein, it is added to various foods as a natural product of high safety and used as a dietary supplement [13]. Absorption of intact LF without degradation from the small intestine is necessary [14] to exert the anti-inflammatory effects of LF. However, most orally administered LF is degraded by stomach juice [15], with only trace amounts of intact LF arriving in the small intestine [16]. Therefore, various delivery systems have been used to prevent LF degradation in the stomach. Liposomal LF, a multilamellar phospholipid vesicle containing a significant amount of LF molecules, is used as an effective delivery system. Ishikado et al. reported that liposomal bovine LF (LbLF) improves the resistance of bLF to digestion by artificial gastric juice [17]. Yamano et al. revealed that oral administration of LbLF significantly reduces alveolar bone resorption induced by lipopolysaccharide (LPS) stimulation through inhibition of TNF-α production [14]. Therefore, we hypothesized that bLF administration may be effective in inhibiting the pathological progression of RA, in which TNF-α plays a key role in the development and progression of inflammation and bone destruction, such as periodontitis. To clarify this issue, we used a representative RA mouse model, SKG. SKG mice with a point mutation in the gene encoding ZAP-70 spontaneously develop Th17-mediated chronic autoimmune arthritis, which immunopathologically resembles human RA [18, 19]. Proinflammatory cytokines such as TNF-α, interleukin 1 (IL-1), and interleukin 6 (IL-6) are abundantly produced in the arthritis joints of SKG mice [18, 20]. Deficiency of TNF-α, IL-1, and IL-6 reduces the incidence and severity of RA in SKG mice, similar to the effects of anti-cytokine therapy in human RA [19].

In the present study, we investigated the effects of orally administered LbLF on the pathological progression of RA in SKG mice and clarified the mechanisms underlying the inhibitory effect of bLF on TNF-α-induced activation of synovial fibroblasts and osteoclastogenesis.

## Materials and methods

### Materials and reagents

**RA mouse model**: Six-week-old female SKG mice, which exhibit chronic autoimmune arthritis similar to human RA (genetic RA mouse model), weighing 20–25 g (N = 36) were purchased from CLEA Japan, Inc. (Tokyo, Japan). **Primary synovial fibroblasts from RA patients (RASFs)**: Two types of RASFs were employed; one was purchased from Articular Engineering (Northbrook, IL, USA), while the other was obtained from the synovium of patients with RA after receiving written informed consent in accordance with a protocol approved by the Institutional Ethical Committee of Hiroshima University (Permission No.: E-668). **Bovine lactoferrin (bLF)**: bLF was purchased from the Morinaga Milk Industry (Tokyo, Japan). **Liposomal lactoferrin (LbLF)**: LbLF, which is composed of multilamellar vesicles, was prepared by hydrating dietary soy phosphatidylcholine with an aqueous solution containing bLF, as described previously [14]. **RA inducer**: Mannan from *Saccharomyces* was obtained from Sigma-Aldrich (St. Louis, MO, USA) and dissolved in 25 mg/mL injection solvent before intraperitoneal injection (Mannan: 20 mg/mice). **Antibodies**: Anti- TNF-**α** rabbit polyclonal antibody (ab9739; Abcam, Cambridge, UK) and bovine lactoferrin mAb a-bC-lobe (Hycult Biotech Inc., PA, USA) were used for immunohistochemistry. **Reagents**: Polyclonal anti-immunoglobulin G (anti-IgG), monoclonal anti-TNF receptor-associated factor 2 (TRAF2), and anti-bLF antibodies were purchased from Santa Cruz Biotechnology (Santa Cruz, CA, USA). Polyclonal anti-phospho-p65, anti-p65, monoclonal anti-phospho-c-Jun N-terminal kinase (anti-phospho-JNK), anti-JNK, anti-phospho-p38, anti-p38, anti-phospho-IκB kinase β (anti-phospho-IKKβ), and anti-IKKβ antibodies were obtained from Cell Signaling Technology (Danvers, MA, USA). **Recombinant human TNF-α**: Recombinant human TNF-**α** (rhTNF-**α**) was purchased from PeproTech, Inc. (East Windsor, NJ, USA).

### *In vivo* experiments

#### Animal experiment

This study was carried out in strict accordance with the recommendations of the Guide for the Care and Use of Laboratory Animals of the Hiroshima University Animal Research Committee and the AVMA Guidelines on Euthanasia. The protocol described below was approved by the Committee on the Ethics of Animal Experiments of Hiroshima University (permit number: A11-47). All mice were housed in a specific pathogen-free facility under 12 h light-dark cycles with access to water and food ad libitum. Intraperitoneal anesthesia was performed using pentobarbital sodium (1.62 mg/30 g; Kyoritsu Seiyaku Co., Tokyo, Japan), midazolam (0.06 mg/30 g; B.W. Sandoz K.K. Tokyo, Japan), and diazepam (0.0045 mg/30 g; B.W. Sandoz K.K.). Animals were always observed after anesthesia until they ambulated normally. Mice were closely monitored for body weight and general health. None of the mice became severely ill during the study, and no unanticipated adverse events occurred. CO_2_ euthanasia, which is commonly accepted for small animals, was used in this study.

S1A Fig shows the experimental schedule. A total of 48 mice were randomly divided into four groups. The first group was the control group (Cont), without any treatments. The second group was the arthritis (Ar) group, which received an intraperitoneal injection of mannan to induce chronic arthritis. The third and fourth groups were Ar+LbLF100 and Ar+LbLF500, which were orally administered 100 and 500 mg/day/kg of LbLF, respectively. LbLF was applied through drinking water for seven consecutive days before mannan injection and was continuously applied throughout the experimental period. Drinking water containing the vehicle of LbLF was supplied to the Cont and Ar groups. The concentrated LbLF solution containing 12% bLF, 4% soy phosphatidylcholine, and 35.3% glycerin was originally made with distilled water. Based on preliminary measurement of drinking water intake, we adjusted the solution to a mouse intake of 100 mg/day/kg or 500 mg/day/kg of bLF. S1B Fig shows the average amount of LbLF in the drinking water. The mice in each group were constantly administered approximately 100 mg/day/kg or 500 mg/day/kg of LbLF.

At 5 weeks (n = 4 for cytokine measurement and n = 3 for measurement of Th17 and regulatory T (Treg) cells in the spleen) and 15 weeks (n = 5) after mannan injection, tissue samples were collected from the hind limb ankle joint with chronic arthritis.

During the experimental period, the water intake, body weight, and macroscopic swelling score of the joint according to Sakaguchi’s criteria were recorded.

#### Tissue preparation

For the preparation of tissue sections, tissue samples were fixed and decalcified as previously described [14]. The samples were embedded in paraffin in the sagittal direction, and serial sections (6.0 μm thick) were made in the direction parallel to the long axis of the legs, including the RA joints. Finally, the sections were stained with hematoxylin and eosin for histological examination.

#### Immunohistochemical analysis

Immunohistochemical staining was performed as previously described [14]. A Dako EnVision+ system (Dako Japan, Tokyo, Japan) was used for immunohistochemical staining. Endogenous peroxidase activity was blocked using 0.3% H2O2 in methanol for 30 min. The sections were then incubated with a protein block serum-free solution (Dako Japan) for 10 min. Anti-TNF-α polyclonal and bovine lactoferrin monoclonal antibodies were used at a dilution of 1:250 or 1:100 and incubated for 2 h or overnight, respectively. The sections were then incubated with labeled polymer-HRP-anti-rabbit or -mouse (Dako Japan) for 1 h at 23–25°C. The color was developed using 0.025% 3,3’-diaminobenzidine tetrahydrochloride in Tris-HCl buffer plus hydrogen peroxide (Dako Japan). The negative controls included phosphate-buffered saline (PBS) or rabbit serum in lieu of the primary antibody.

#### Gene expression

The skin on the ankle joint of the right hind limb was removed. The soft tissue surrounding the joint was placed in liquid nitrogen and stored at −80°C until analysis. Total RNA was extracted from the pannus and bones of the diseased joints using TRIzol reagent (Invitrogen, Waltham, MA, USA). One microgram of total RNA was used for cDNA synthesis using a ReverTra Ace kit (Toyobo, Osaka, Japan). Quantitative real-time reverse transcription PCR was performed in an Applied Biosystems Step One Plus real-time PCR system (Applied Biosystems, Waltham, MA, USA) using a TagMan Fast Universal PCR Master Mix (Applied Biosystems) and specific primers and probes for TNF-α (Applied Biosystems; Forward: 5’-CCAAATGGCCTCCCTCTCAT-3’, Reverse: 5’-GCTACAGGCTTGTCACTCGAAATT-3’, TagMan probe: 5’-CCCAGACCCTCACACTCAGATCATCTTC-3’) (1). The reaction product was quantified using glyceraldehyde 3-phosphate dehydrogenase (Mm99999915_g1; Applied Biosystems) as the reference gene.

#### CT imaging

Bone destruction of the right or left hind limb ankle joint from each group was evaluated by micro-computed tomography (μ-CT) using a Skyscan (TOYO Corporation, Tokyo, Japan) with X-ray tube settings of 90 kV and 108 μA. Two hundred projections were scanned at a voxel size of 15.0 μm. NRecon (Bruker microCT, Antwerpen, Belgium) and CTbox software (Bruker microCT) were used to generate three-dimensional models and perform bone morphometry, respectively.

#### Flow cytometry detection of Treg/Th17 cells

The spleen was removed from each experimental group of mice, and an appropriate amount of spleen cells was collected for fluorescent-labeled antibody staining and analysis of Th cell subsets by flow cytometry.

Briefly, the mincing spleen was gently crushed with a syringe, and a single-cell suspension was prepared by filtration through a 70 μm cell strainer. To remove erythrocytes, hemolysis was performed using ammonium chloride [NH_4_Cl (Sigma-Aldrich)] on ice for 5 min. After stopping the hemolysis reaction, the number of cells was counted and seeded on a 12-well culture dish at a density of 4 × 10^6^ cells/well and treated with 20 ng/mL phorbol 12-myristate 13-acetate (PMA) and 1 μg/mL ionomycin (Santa Cruz Biotechnology) in complete media for 6 h. Three hours after stimulation, Golgi stop (BD Biosciences, Franklin Lakes, NJ, USA) was added at a final concentration of 1.6 μM. After an additional 6 h of cultivation, the cell suspension was washed with PBS containing 2% fetal bovine serum (FBS) and divided into two groups: a CD3 + CD4 + Th17 analysis group and a CD3 + CD4 + Treg analysis group.

Cells were stained with Fixable Viability dye eFluor450 (eBioscience, San Diego, CA, USA), FITC anti-CD3 antibody (eBioscience), and APC anti-CD4 antibody (eBioscience) at 4°C for 30 min. After membrane fixation and permeation using a Foxp3/Transcription Factor Staining Buffer Set (eBioscience), cells were stained with PE anti-IL-17A antibody (eBioscience) or PE anti-Foxp3 antibody (eBioscience) at 4°C for 30 min. The samples were analyzed by flow cytometry using a fluorescence-excited cell separation analyzer (Cell Analyzer EC800; Sony, Tokyo, Japan).

### *In vitro* experiments

#### Cell culture

Cultured cells from the first to the sixth passage were used for the present experiments. Cells were maintained in Dulbecco’s Modified Eagle Medium (DMEM) (Nissui Pharm, Tokyo, Japan) with 10% FBS (Invitrogen) and 100 units/mL penicillin-streptomycin (Invitrogen) at 37°C in a humidified atmosphere containing 5% CO_2_.

#### TNF-α-induced osteoclast formation in primary bone marrow cells

Mouse bone marrow cells (BMCs; 2×10^3^ cells) were seeded, and 2 days later, TNF-α (50 ng/mL) and bLF (0, 1, 10, and 100 μg/mL) were added and cultured for an additional 5 days. The same treatment was performed for the negative control (control group) without any treatment. Cultured cells were fixed and stained for TRAP, an osteoclast marker enzyme.

#### Determination of cytokine production by ELISA

RASFs were seeded in a 24-well culture plate at an initial density of 5 × 10^4^ cells/well and cultured as described above. After 4 h of pretreatment with bLF (1, 10, and 100 μg/mL), cells were washed with PBS. The cells were then incubated with rhTNF-α (10 ng/mL) for 48 h, and the culture media were collected.

Protein levels in the culture media were measured using an ELISA kit (Human TNF-α DuoSet; R&D Systems, Minneapolis, MN, USA) according to the manufacturer’s instructions.

#### Lipoprotein receptor related protein-1 siRNA gene knockdown

Lipoprotein receptor related protein-1 (LRP1) siRNA oligomers and control siRNA were purchased from Applied Biosystems. RASF cells were transfected with siRNA when they reached 30%–50% confluence in 10% DMEM without antibiotics, according to the manufacturer’s protocol. Briefly, gene-specific LRP1 siRNA oligomers (50nM) were diluted in 100 μL Opti-MEM I reduced serum medium (Opti-MEM; Invitrogen) and mixed with 4 μL of lipofectamine RNAiMAX transfection reagent (Invitrogen) pre-diluted in 96 μL Opti-MEM. After 20 min of incubation at 22–23°C, the complexes were added to the cells at a final volume of 2 mL medium. The medium was replaced with fresh 10% DMEM with antibiotics after 6 h.

#### Western blot and immunoprecipitation analyses

RASFs were seeded in a 3.5 cm culture dish at an initial density of 2.5 × 10^5^ cells/dish. Four hours after pretreatment with bLF (1, 10, and 100 μg/mL), the RASFs were washed with PBS, incubated with rhTNF-α (10ng/mL) for 15 min, and then collected. Western blotting was performed as described previously [21]. Briefly, the collected cells were lysed in ice-cold lysis buffer containing 50 mM Tris-HCl (pH 7.5), 250 mM NaCl, 0.1% Triton X-100 (Roche, Basel, Switzerland), 1 mM EDTA, 50 mM NaF, 0.1 mM Na3VO4, 1 mM DTT, 0.1 mM leupeptin, 0.1 μg/mL soybean trypsin inhibitor, 10 μg/mL L-1 chlor-3-(4-tosylamido)-4-phenyl-2-butanon, 10 μg/mL L-1 chlor-3-(4-tosylamido)-7-amino-2-heptanon-hydrochloride, 10 μg/mL aprotinin, and 50 μg/mL phenylmethylsulfonyl fluoride. Protein concentration was determined by Bradford protein assay (Bio-Rad Laboratories, Hercules, CA, USA) using bovine serum albumin (Sigma-Aldrich) as a standard. Next, 20 μg of protein lysate was subjected to 10% polyacrylamide gel electrophoresis, followed by electroblotting onto a nitrocellulose filter. An ECL western blotting detection system (Amersham Biosciences, Amer) was used to detect the immunocomplex. The immunoprecipitates were also subjected to western blotting.

#### Statistics

SSRI for Windows (Social Survey Research Information Co., Ltd., Tokyo, Japan) was used for statistical analysis. The experiments were performed in triplicate. Data are presented as mean ± standard deviation. Statistical differences among experimental groups were evaluated by one-way analysis of variance followed by Tukey’s post-hoc test, with the level of significance set at p < 0.01 (**) and p < 0.05 (*).

## Results

### Orally administrated LbLF prevented joint swelling and bone destruction in SKG mice

There was no difference in body weight among the experimental groups (S1C Fig), indicating that orally administered LbLF had no harmful effects on mouse health.

**Fig 1:**
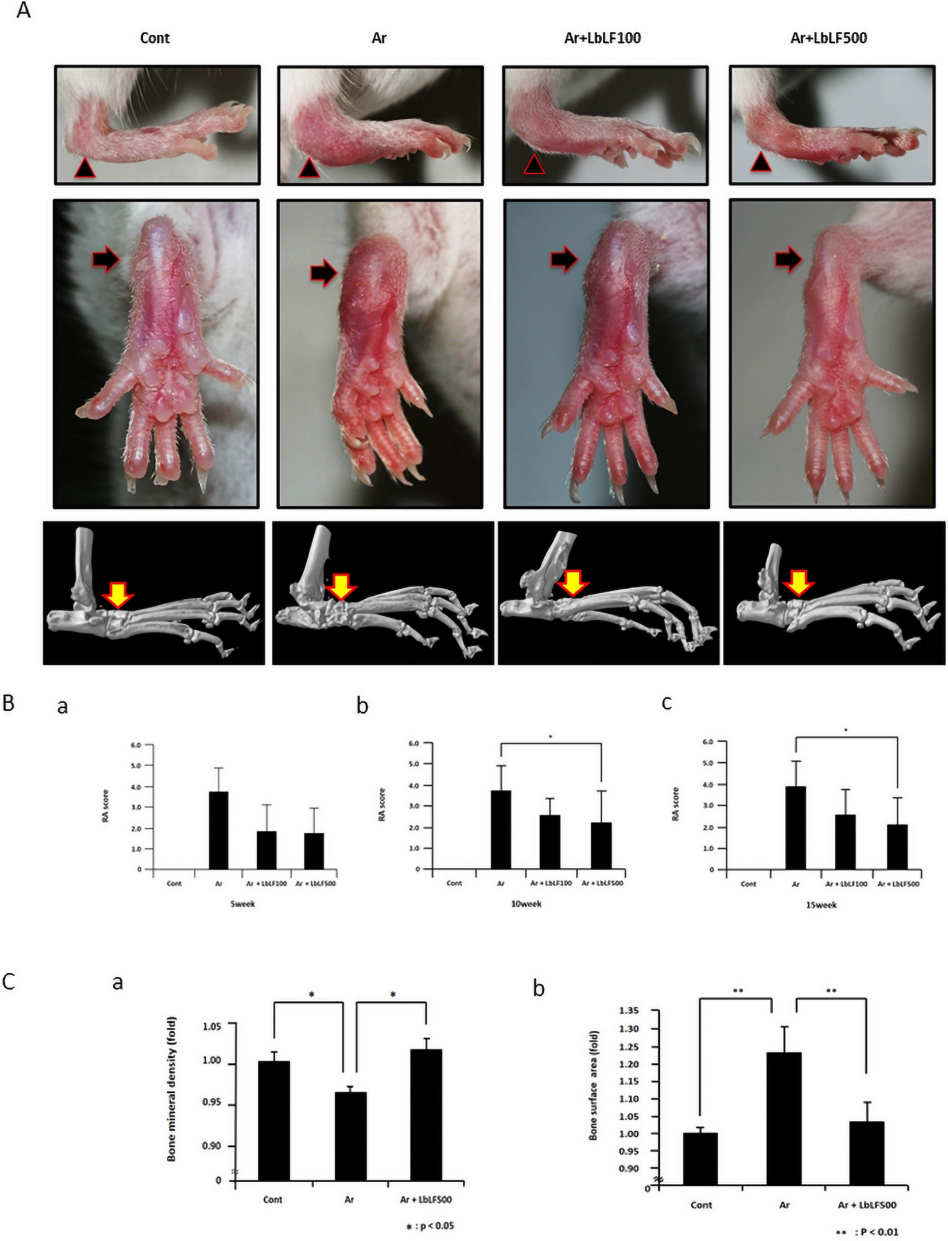
Orally administered liposomal bovine lactoferrin (LbLF) markedly reduced swelling and bone destruction of leg joints. (A) Macroscopic and micro-computed tomography findings at 15 weeks after arthritis (Ar) induction. Redness and swelling of hindpaw ankles (black arrow heads and arrows) in the Ar group were prominent in comparison with those in the control (Cont). LbLF application suppressed redness and swelling in a dose-dependent manner. LbLF application prevented bone destruction (yellow arrow) caused by mannan injection. (B) Rheumatoid arthritis (RA) score. RA scores in the Ar+LbLF500 group at 10 and 15 weeks were significantly lower than those in the Ar group. N = 5, * = P<0.05. (C) Bone mineral density. LbLF500 treatment mitigated the reduction in bone mineral density caused by mannan injection. * = P<0.05. Bone surface area. In the Ar group, mannan injection induces a significant increase in the bone surface area. In the LbLF treated animals, this increase in bone surface area was markedly prevented. ** = P<0.01.

Macroscopically, a reduction in leg joint redness and swelling in the LbLF-treated groups was observed (Fig 1A). Fig 1Ba–1Bc shows the time-dependent changes in leg joint swelling measured by the Sakaguchi method [18] during the experimental period; none of the mice in the control group had leg joint swelling (RA score = 0). The score of the Ar group gradually increased to3.85±0.92 at 5 weeks after mannan injection and remained relative stable until 15 weeks, with a slight increase. Conversely, RA scores in the Ar+LbLF100 and Ar+LbLF500 groups slowly increased to 1.98±1.15 and 1.82±1.22 at 5 weeks, 2.62±0.71 and 2.22±1.45 at 10 weeks, and 2.56±1.27 and 2.11±1.34 at 15 weeks, respectively. Orally applied LbLF inhibited leg joint swelling. In particular, the Ar+LbLF500 group showed significant suppression at 10 and 15 weeks (P<0.05).

μ–CT examination showed severe bone destruction in the diseased joint 15 weeks after arthritis induction with mannan. In the Ar+LbLF100 and Ar+LbLF500 groups, joint bone destruction was slight, indicating the effective inhibition of bone destruction with LbLF application (Fig 1A, yellow arrows). Fig 1Ca shows the bone mineral density of the calcaneus. The bone mineral density in the Ar group was significantly lower than that in the control group (p<0.05). Oral administration of LbLF500 markedly improved the reduction in bone mineral density (p<0.05). In contrast, the bone surface area of the Ar group was higher than that of the control group (P<0.01), and oral administration of LbLF500 significantly suppressed the increase in surface area caused by mannan injection (P<0.01) (Fig 1Cb).

### Orally applied LbLF suppressed inflammation in the diseased joint

**Fig 2:**
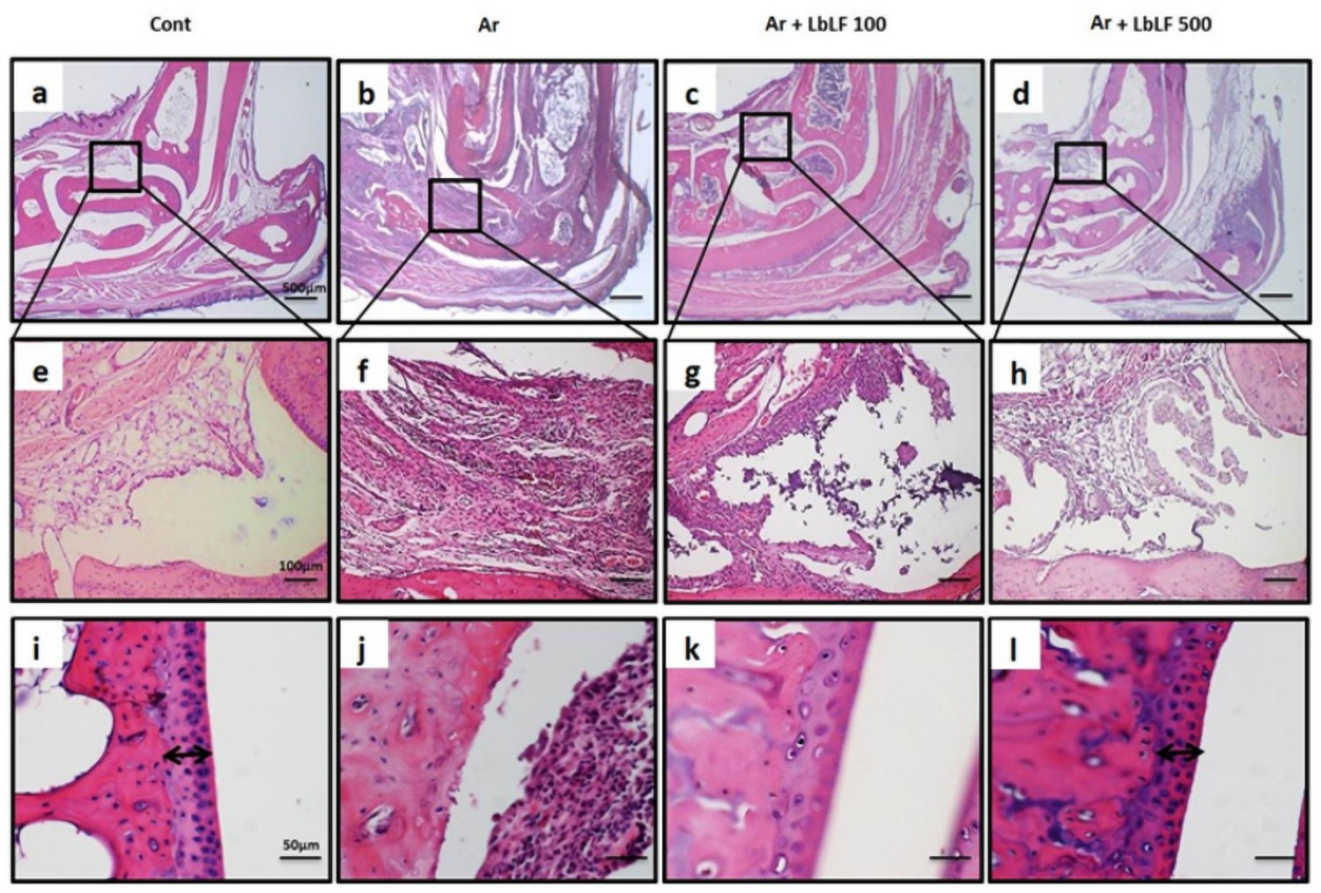
Orally administered liposomal bovine lactoferrin (LbLF) suppressed inflammation in diseased leg joints at 15 weeks after mannan injection. Leg joint histology. In the arthritis (Ar) group, prominent pannus proliferation (b, f) was observed, and the joint cartilage layer was completely destroyed (j) comparing to those in the control (Cont) (a, e, i). LbLF100 (c, g, k) and LbLF500 (d, h, l) application alleviated pannus formation and cartilage destruction.

Fifteen weeks after arthritis induction, the leg joint histologically showed proliferation of synovial cells with inflammatory cell infiltration; the so-called pannus formation was prominently seen in the Ar group (Fig 2b and 2f). The joint cartilage layer observed on the joint bone surface in the control group (Fig 2i) was completely destroyed in the Ar group (Fig 2j). In contrast, in the Ar+LbLF100 (Fig 2c and 2g) and Ar+LbLF500 groups (Fig 2d and 2h), inflammatory cell infiltration, synovial cell proliferation, and bone destruction were reduced in comparison with those in the Ar groups (Fig 2b and 2f). Moreover, the cartilage layer on the surface of the joint bone (Fig 2k and 2l) was conserved in the control group (Fig 2i).

### Orally applied LbLF reduced expression of TNF-α in the pannus of SKG mice

**Fig 3:**
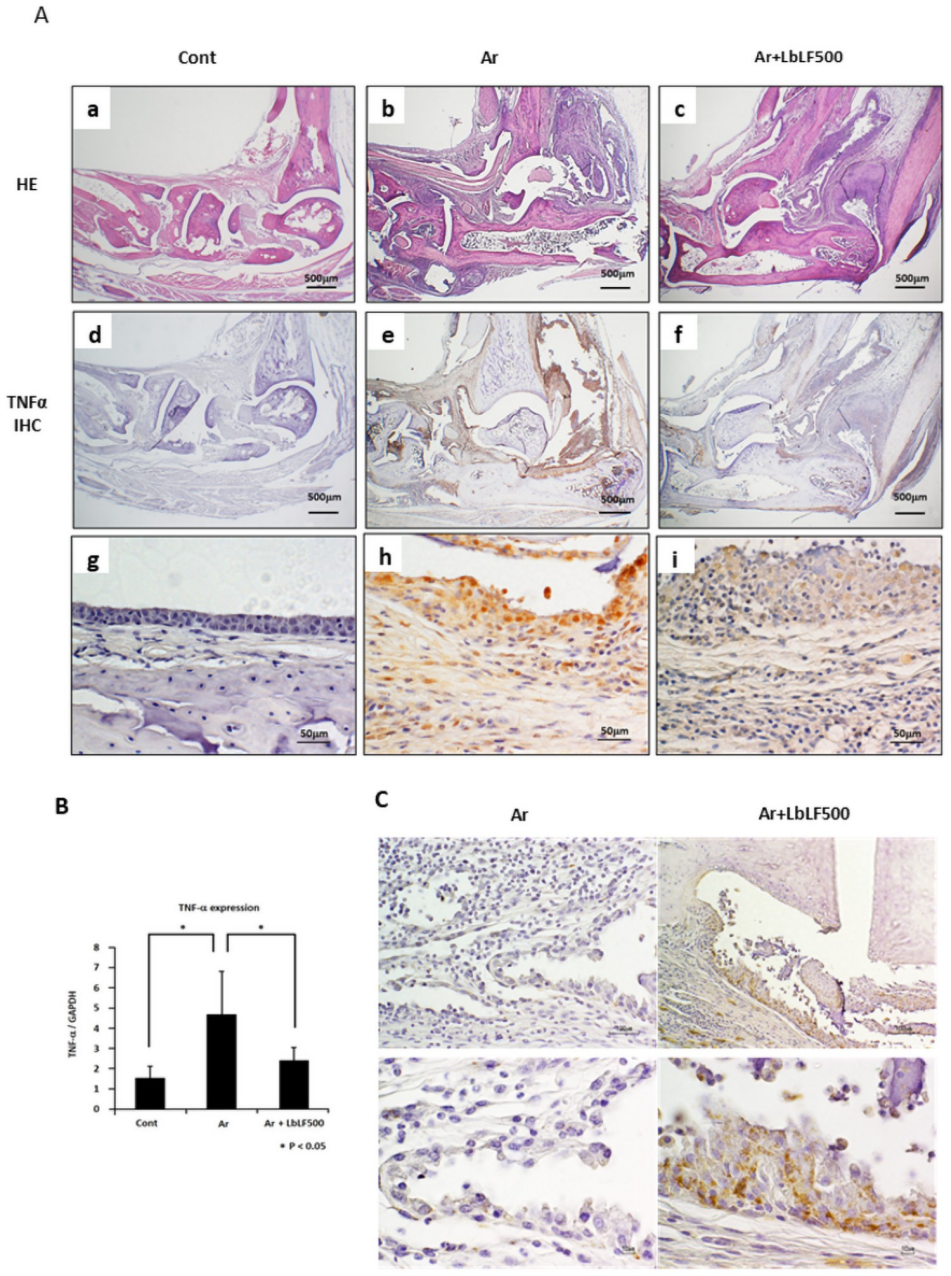
Orally administered liposomal bovine lactoferrin (LbLF) reduced expression of tumor necrosis factor α (TNF-α) in pannus. (A) Immunohistochemistry of TNF-α. In the arthritis (Ar) group (Ab, e, h), intense immune-expression of TNF-α was widely observed in the pannus compared to that in the control group (Cont) (Aa, d, g). Reduced expression was evident in the LbLF500 administered group (Ac, f, i). (B) TNF-α mRNA measured by real time PCR and normalized to glyceraldehyde 3-phosphate dehydrogenase (GAPDH) mRNA. The TNF-α mRNA level was significantly higher than that in Cont. A remarkable reduction in the TNF-α level was observed in the Ar+LbLF500 group. N = 4, * = P<0.05. (C) Immunohistochemistry of bLF. Immunolocalization of bLF was observed in synovial cells and macrophages in the pannus of the Ar+LbLF500 mice but not in that of the Ar mice.

As TNF-α plays a critical role in the exacerbation of inflammation and bone destruction in RA [5], TNF-α expression in the pannus of the ankle joint was immunohistochemically examined. In the control group, TNF-α-positive cells were not observed (Fig 3Ad and 3Ag). Intense positivity for TNF-α was extensively observed in the widely formed pannus around the ankle joint of the Ar mice (Fig 3Ae). Fig 3Ah shows a high-power view of the affected ankle joint in the Ar group. Intense positive reactivity was observed in synovial lining fibroblasts and macrophages in the proliferated pannus. Conversely, orally administered LbLF, especially LbLF at 500 mg/day/kg, significantly reduced the staining intensity and number of TNF-α–positive cells in the pannus (Fig 3Af and 3Ai).

Compared to that in the control group, the level of TNF-α mRNA in the Ar group (Fig 3B) was significantly increased (p<0.05). The level of TNF-α mRNA in the Ar+LbLF500 group was significantly downregulated (p<0.05). Fig 3C shows immunolocalization of bLF. Only in Ar+LbLF500, an intense positive reaction for bLF, which was administered orally, was detected in synovial cells and macrophages in the pannus of the diseased joints.

### Orally applied LbLF improved the imbalance of Th17 cells and regulatory T-cells in the spleen

**Fig 4:**
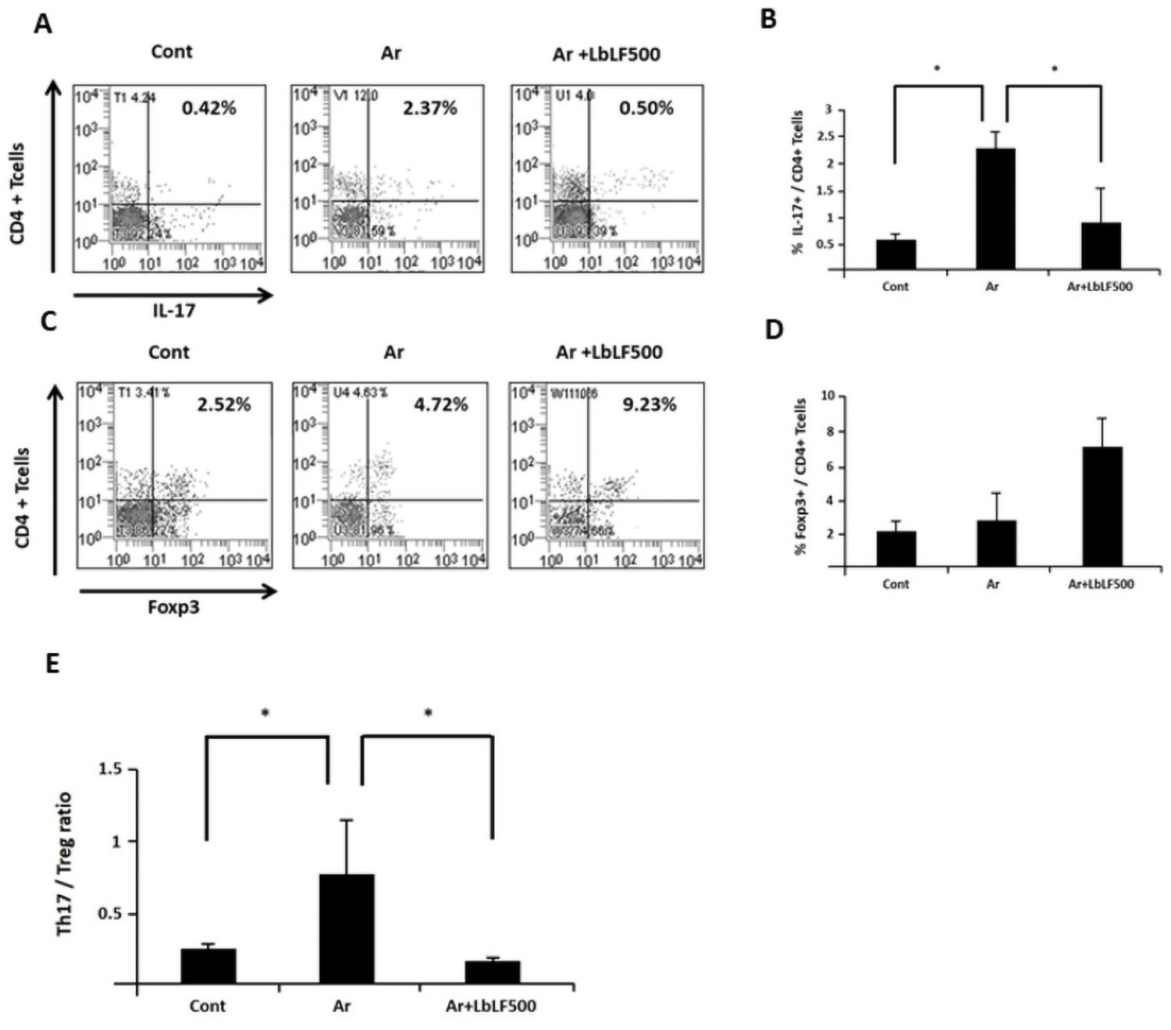
Orally administered liposomal bovine lactoferrin (LbLF) improved the imbalance of Th17 cells and regulatory T-cells in the spleen. (A,B) Percentage of Th17 cells in the spleen. Orally administered LbLF significantly reduced the percentage of Th17 cells increased in Ar group. (C,D) Percentage of Treg cells in the spleen. The number of Treg cells tended to increase in the arthritis (Ar)+LbLF500 group. (E) Th17 / Treg cell ratio. This ratio was significantly upregulated in the Ar group (P <0.05), whereas it significantly improved in the Ar + LbLF 500 group (P <0.05).

The number of Th17 cells in the spleen increased from 0.42% in the control group to 2.37% in the Ar group. In contrast, the Ar + LbLF 500 group contained 0.5% (Fig 4A). The increased proportion of Th17 cells/CD4+ T cells in the Ar group was significantly reduced by the oral administration of LbLF (Fig 4B). The number of Treg cells in the spleen was 2.52%, 4.72%, and 9.23% in the control, Ar, and Ar + LbLF 500 group, respectively. Oral administration of LbLF increased the proportion of Treg cells (Fig 4C and 4D). Fig 4E shows the Th17/T reg cell ratio. The Th17/Treg cell ratio, which was significantly increased in the Ar group (P <0.05) compared to that in the control, also significantly improved in the Ar + LbLF 500 group (P <0.05).

### bLF inhibited TNF-α production caused by TNF-α stimulation

**Fig 5:**
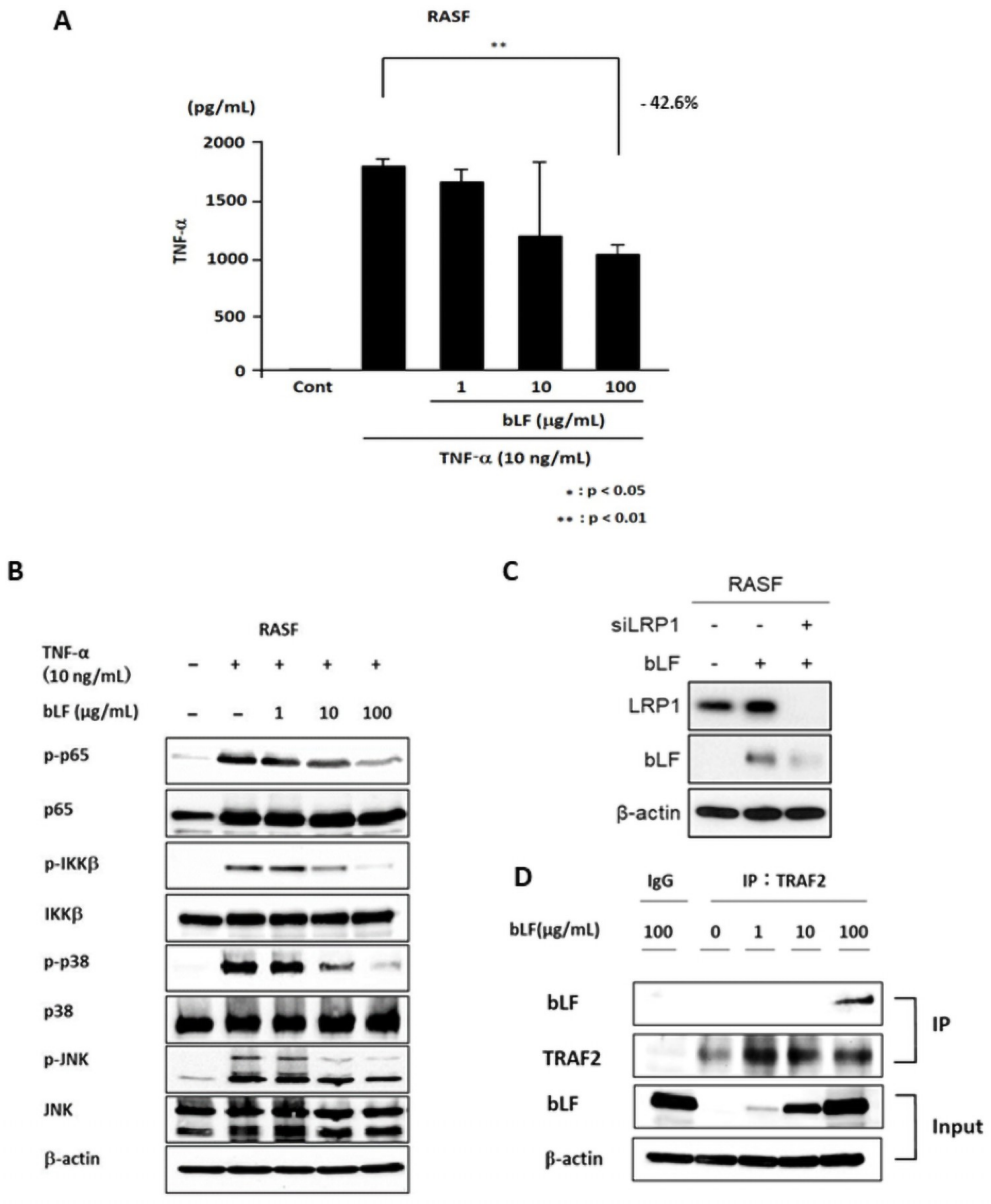
Bovine lactoferrin (bLF) inhibited tumor necrosis factor α (TNF-α) production from synovial fibroblasts from rheumatoid arthritis patients (RASFs) with TNF-α stimulation by binding to TNF receptor-associated factor 2 (TRAF2). (A) TNF-α production by RASFs was analyzed by ELISA. The results showed that 100 μg/mL bLF induced a 42.6% reduction in TNF-α secretion. (B) Activation of nuclear factor κB and mitogen-activated protein kinase signaling in TNF-α-stimulated RASFs was observed. bLF downregulated p-p65, p-c-Jun N-terminal kinase, and p-p38 expression. (C) LRP-1 was knocked down by siRNA and cultured for 24 h, then the cells were treated with 10 μg/mL bLF for 24 h. bLF expression was reduced in siLPR-1 RASF cells. (D) RASF cells were treated with 1, 10, or 100 μg/mL bLF for 4 h. Whole cell lysates were immunoprecipitated with anti-TRAF2 and examined by immunoblotting with bLF. Immunoblotting of TRAF2 and bLF was performed to control immunoglobulin G for protein loading. TRAF2-immunoprecipitated protein showed bLF by immunoblotting. bLF expression in whole cell lysate (input) was also analyzed by immunoblotting. bLF increased in a dose-dependent manner. The experiments were performed in duplicate with similar results.

TNF-α is mainly produced by synovial fibroblasts and macrophages [21]. We examined the effects of bLF on TNF-α production from TNF-stimulated RASFs by ELISA. A significant 42.6% reduction in TNF-α in the culture medium containing RASFs with TNF-α stimulation (Fig 5A) was observed in the 100 μg/mL bLF pretreatment culture (p<0.01).

### bLF downregulated TNF-α-induced signaling pathways through binding to TRAF2

TNF-α can induce nuclear factor kappa B (NF–κB) and mitogen-activated protein kinase (MAPK) activation [1]. The effects of bLF on the NF-κb and MAPK signaling pathways activated by rhTNF-α stimulation were examined. bLF inhibited phospho-p65 and phospho-IKKβ of the NF-κB pathway in RASFs with TNF-α stimulation (Fig 5B). bLF also suppressed the phosphorylation of p38 and JNK in the MAPK pathway.

bLF, which is mainly endocytosed by LRP1, inhibits LPS-activated NF-κB and MAPK pathways by binding to TRAF6, an adaptor protein ubiquitin ligase [21]. TRAF2, which is upstream of IKKβ, contributes to the activation of MAPK and NF-κB signaling caused by TNF-α. We hypothesized that the TNF-α-activated signaling pathway may be inhibited by bLF binding to TRAF2.

LRP-1 knockdown RASF cells with bLF treatment showed a marked reduction of intracytoplasmic bLF in comparison with that in the control RASFs, indicating that bLF was mainly internalized into RASF cells through LRP-1 (Fig 5C). Internalized bLF increased in an applied bLF dose-dependent manner (Fig 5D; bLF in the input lane). TRAF2-immunoprecipitated proteins included bLF (Fig 5D; bLF and TRAF2 in input lanes). It has been suggested that exogenously applied bLF enters the cytoplasm and binds directly to TRAF2 or somewhere in the TRAF2-TRADD-RIP complex in RASF cells.

### bLF reduced osteoclast formation from primary BMCs caused by TNF-α

**Fig 6:**
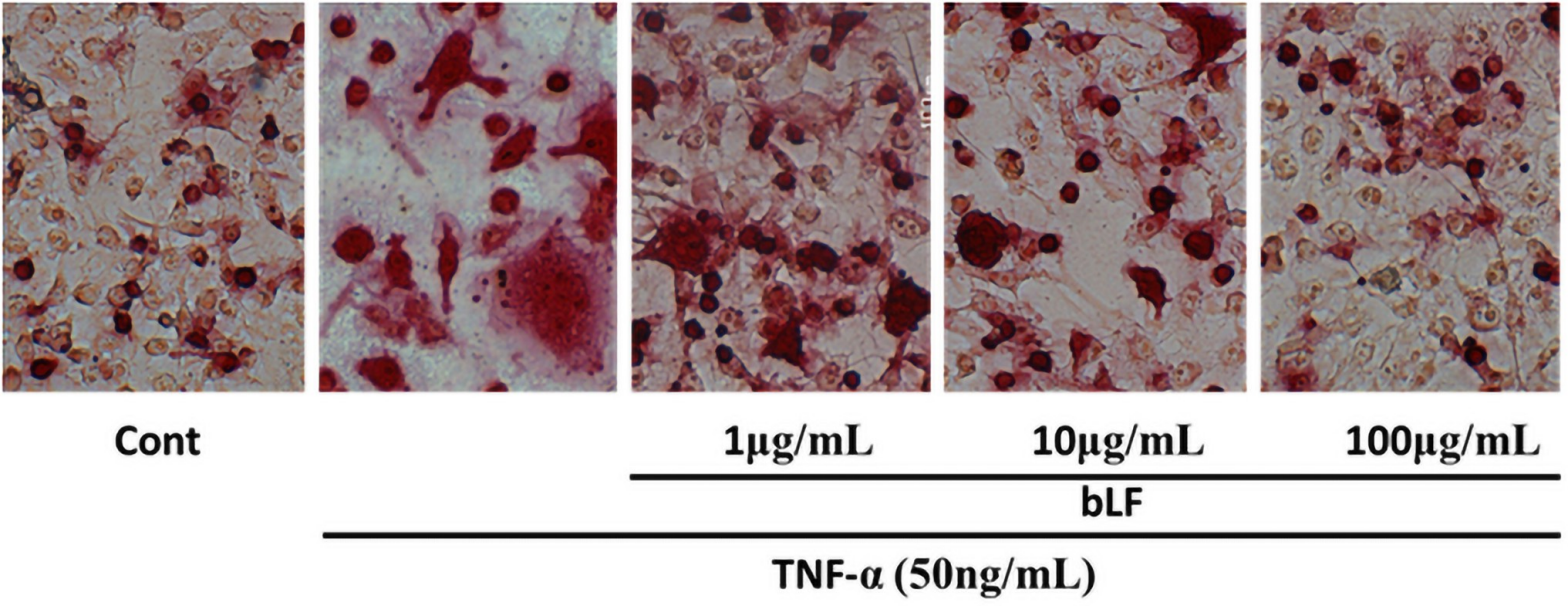
Bovine lactoferrin (bLF) reduced osteoclast formation from primary bone marrow cells (BMCs) caused by tumor necrosis factor α (TNF-α) stimulation. Large multinucleated giant osteoclasts were formed in TNF-α -stimulated primary BMC cultures. Treatment with bLF markedly decreased the number of TRAP-positive osteoclasts, and the size of osteoclasts was smaller than that of TNF-α-stimulated osteoclasts.

A single culture of primary BMCs was used to confirm the direct effect of bLF on TNF-α-induced osteoclastogenesis. Large multinucleated giant osteoclasts were formed in TNF-α-stimulated primary BMC cultures. Treatment with bLF markedly decreased the number of TRAP-positive osteoclasts, and the size of the osteoclasts was smaller than that of the TNF-α-induced osteoclasts (Fig 6).

## Discussion

In the present study, we used SKG mice, in which a spontaneous point mutation of the gene encoding the SH2 domain of ZAP-70, a key signal transduction molecule in T cells, might cause chronic autoimmune arthritis similar to human RA [18]. However, spontaneous onset of RA in SKG mice is quite rare under specific-pathogen-free conditions. Therefore, mannan was injected intraperitoneally as an RA inducer. Mannan activates a complement component, C5a, and promotes the production of inflammatory cytokines, such as TNF-α and IL-6, from macrophages and synovial fibroblasts, followed by differentiation and activation of CD4^+^Th17 cells in synovial tissue [1, 22], finally leading to early onset of RA in SKG mice. In the present study, 3 weeks after Mannan injection, all SKG mice showed RA-like features in the finger, wrist, and ankle joints of the forepaws or hindpaws. Bone destruction of the finger and ankle joints was observed using μ–CT. Moreover, histological pannus formation showing synovial fibroblast proliferation, and lymphocyte and macrophage infiltration was seen in the peri-joint area, similar to that of human RA. Thus, the usefulness of SKG mice as an animal model of RA was confirmed.

In the present study, RA-induced SKG mice were orally pretreated with LbLF 7 days before mannan injection; LbLF was also continuously administered throughout the experimental period. Orally administered LbLF inhibited redness and swelling of the forepaw joints and arthritis, including pannus formation, cartilage erosion, and bone destruction. TNF-α expression was remarkably reduced in the pannus of the Ar+LbLF500 group. In RA, the proliferation of synovial fibroblasts and joint bone/cartilage destruction are induced by pro-inflammatory cytokines such as TNF-α, IL-1β, and IL-6 produced by synovial fibroblasts and macrophages in the pannus. In particular, TNF-α plays an important role in the cytokine network and promotes both the production of other cytokines and osteoclastic bone resorption [23, 24]. Yamano et al. reported that using an LPS-induced periodontitis mouse model, orally administered LbLF inhibits LPS-induced alveolar bone destruction through the inhibition of TNF-α production in periodontal tissue [14]. In our study, oral administration of LbLF significantly inhibited joint bone destruction by reducing TNF-α production in the pannus. Moreover, using a single culture of BMCs, TNF-α-induced osteoclastogenesis from BMCs was remarkably inhibited by application of bLF (Fig 4). These results suggest that LbLF has a beneficial impact on preventing the onset and pathological progression of RA.

The LbLF used in the present study included large amounts of bLF in multilayers of liposomes to avoid degradation by stomach enzymes and to increase the amount of intact bLF entering the intestinal tract. Ishikado et al. reported that the amount of TNF-α produced by LPS stimulation from peripheral blood mononuclear cells isolated from mice after 7 days of oral administration of LbLF is reduced by 1/4 in LbLF-administered mice compared to that in the control group. This indicates the effectiveness of liposomes as a delivery system [17]. Yuan et al. reported that 77.9% of solid lipid nanoparticles (SLNs), such as liposomes, are taken from the intestine and circulated *in vivo* through the lymphatic system. The rest of the SLNs are directly taken into the blood through paracellular transport or capillaries [25]. Talukder et al. reported that LF receptors are distributed in the small intestinal and colonic epithelium, especially in Peyer’s patches [26]. Yamano et al. confirmed the localization of bLF in Peyer’s patches and the small intestinal epithelium in LbLF-treated rats [14]. Many lymphocytes and macrophages in Peyer’s patches are positive for bLF. Furthermore, bLF has been detected in various organs, including the spleen, bone, and periodontal tissue, indicating that bLF is absorbed from the intestinal tract and disseminated to the whole body through blood and/or lymphatic circulation. In the present study, immunolocalization of orally administered bLF was detected in the cytoplasm of synovial cells and macrophages in the pannus of the Ar+LbLF500 group but not in that of the Ar group. In our animal model, LbLF was freely taken from the drinking water. Therefore, it is considered that low doses of bLF were continuously absorbed from the small intestine, delivered to arthritis-affected joints through lymphatic and blood circulation, and internalized in synovial fibroblasts and macrophages, resulting in significant downregulation of the cytokine burst from these cells. However, further studies are needed to clarify bLF dynamics *in vivo*.

Recently, it has been suggested that dysbiosis of intestinal bacterial flora is a potent pathogenesis and/or exacerbating factor in RA. Several global analyses of fecal bacteria in patients with RA have been performed [27–29]. These studies clarified that the presence of *Bifidobacterium*, *Bacteroides fragilis*, *Bacteroides*, *Porphyromonas*, *Prevotella*, *Eubacterium rectale*, and *Clostridium coccoides* in RA patients is less than that in fibromyalgia. bLF increases intestinal *Bifidobacterium* and regulates intestinal functions [30]. Therefore, it is conceivable that oral administration of LbLF may improve the intestinal flora of patients with RA and inhibit the pathological progression of RA. Thus, it is necessary to examine the effect of orally administered LbLF on the intestinal bacterial flora.

As TNF-α plays an important role in the cytokine network involved in the development and progression of RA, we focused on the effects of bLF on TNF-α-induced inflammatory signaling pathways. TNF-α binds to TNFR1 on the cell membrane and promotes various inflammatory cytokines via inflammatory signaling [31]. Two transcription factors, NF-κB of the NF-κB pathway and AP-1 of the MAPK pathway, play an important role in the expression of inflammatory cytokines through TNFR1. Activated NF-κB (p-p65) is then transferred to the nucleus. In contrast, intranuclear AP1 is activated by phosphorylation of JNK and p38 in the MAPK pathway [32]. Both transcription factors induce production of various inflammatory cytokines. TRAF2, an adaptor protein/ubiquitin ligase, plays a critical role in both NF-κB and MAPK signaling by regulating RIP ubiquitination [33, 34]. In the present study, bLF inhibited the phosphorylation of IKKβ, p65, JNK, and p38, which are downstream of the TNF-α-induced inflammatory signaling pathway, and suppressed TNF-α production from synovial fibroblasts. Interestingly, bLF bound to TRAF2 immunoprecipitated protein, which might include not only TRAF2 but also TRADD and RIP1, from synovial fibroblasts. In addition to synovial fibroblasts, we confirmed that bLF was internalized by PMA-treated THP-1 macrophages and inhibited cytokine production by binding to TRAF2, similar to RASFs (S2 Fig). Therefore, we considered that bLF bound to TRAF2 directly or indirectly, and then inhibited TNF-α-induced inflammatory signals by interrupting polyubiquitination of TRAF2 through inhibition of RIP ubiquitination. Moreover, we confirmed that exogenously applied bLF mainly internalized synovial fibroblasts via LRP-1. It is well known that bLF binds to the LRP1 receptor and is internalized within cells through clathrin-mediated endocytosis [35, 36].

Although we mainly focused on TNF-α signaling as a functional mechanism in the present study, other cytokines such as IL-6 and IL-17 are also important causative factors of RA. We confirmed that IL-17 secretion from cultured RASFs with TNF-α stimulation was reduced by bLF treatment (S3A Fig). In addition, IL-17 secretion by isolated spleen cells obtained from the Ar+LbLF mice significantly decreased in a dose-dependent manner (S3B Fig). This evidence indicates the possibility that bLF can inhibit not only TNF-α but also other critical cytokines, such as IL-17 and IL-6. In particular, IL-17 downstream signaling includes TRAF2, TRAF5, and TRAF6 [37], suggesting that bLF may inhibit the harmful action of IL-17 by binding to them. Inubushi et al. examined the effect of bLF on TNF-α production from osteoblasts stimulated by LPS and clarified that internalized bLF bound to TRAF6, which is a member of the TNF receptor-associated factor protein family, inhibits autoubiquitination of TRAF6 and suppresses TNF-α production by inhibiting the NF-κB pathway, which is activated by LPS [21]. The binding between bLF and TRAF5/Traf6 in RASFs should be examined. Moreover, the mechanism by which internalized bLF escapes endosomes and then binds to TRAF2/TRAF5/TRAF6 has not been elucidated. Further studies are required to confirm this hypothesis.

In addition to the importance of cytokines, it has been reported that the onset of RA involves an imbalance between Th17 cells, which promote the autoimmune response, and Treg cells, which suppress the autoimmune response [38]. Hirota et al. reported that in SKG mice, autoimmune arthritis, similar to human RA, spontaneously develops as a result of the adverse roles of Th17 cells [20]. Th17 cells produce IL-17, which has bone resorption activity. IL-17 induces RANKL expression in osteoblasts and synovial fibroblasts and is involved in bone destruction in RA joints [39]. In the present study, oral administration of LbLF to SKG mice significantly reduced the number of Th17 cells, which was increased in the spleen of the Ar group, while the proportion of Treg cells increased, resulting in an improvement in the ratio of Th17 cells/Treg cells. However, the proportion of Treg cells in the Ar group was higher than that in the control group. It has been reported that Treg cells suppress the onset and progression of autoimmune diseases, such as RA [40]. Therefore, there is a possibility that the biological defense mechanism, which prevented an imbalance between Th17 cells and Treg cells in the Ar group by increasing the proportion of Treg cells, was involved as well. As mentioned above, LbLF administration reduced IL-17 production in Ar+LbLF spleen cells. Therefore, oral administration of LbLF not only improves the imbalance between Th17 cells and Treg cells to control the onset of RA but also reduces Th17 production, which causes osteoclast activation, resulting in suppression of bone destruction and prevention of RA progression.

## Conclusion

Orally administered LbLF effectively prevented the pathological progression of RA in a mouse model by suppressing TNF-α production in the pannus. Moreover, bLF reduced TNF-α secretion from synovial fibroblasts by binding to TRAF2. In addition, LbLF administration improved the imbalance in the Th17 cell/Treg cell ratio in arthritis in SKG mice. Oral administration of LbLF may have a beneficial impact on prevention and therapy for RA as a dietary supplement, which can be freely consumed by patients with RA.

## Supporting information

S1 Fig (A) Diagram of the experimental treatments. (B) Average amount of bovine lactoferrin (bLF) obtained from drinking water in each experimental group. (C) Body weight. There were no significant differences between the experimental groups. Ar: Mannan-induced arthritis group. Ar+LbLF100: 100 mg/kg/day liposomal bLF (LbLF)-administered Ar group. Ar+LbLF500: 500 mg/kg/day LbLF-administered Ar group.
(TIF)

S2 Fig Effects of bovine lactoferrin (bLF) on tumor necrosis factor α (TNF-α)-induced TNF-α production and signal transduction in phorbol 12-myristate 13-acetate-treated THP1 macrophages following lipopolysaccharide stimulation. (A) TNF-α production from THP1 cells induced by 10 ng/mL TNF-α was measured by ELISA 4 h after pretreatment with bLF (0, 1, 10, and 100 μg/mL). (B) At 4 h after bLF pretreatment (0, 1, 10, and 100 μg/mL), THP1 cells were stimulated with TNF-α (10 ng/mL). After 15 min, p-p65, p65, p-IκB kinase β (p-IKKβ), IKKβ, p-p38, p-c-Jun N-terminal kinase (p-JNK), and JNK were detected by western blotting. β-actin was used as an internal control. (C) THP1 cells were treated with 1, 10, or 100 μg/mL bLF for 4 h. Whole cell lysates were immunoprecipitated with anti-TNF receptor-associated factor 2 (TRAF2) and examined by immunoblotting with bLF. Immunoblotting of TRAF2 was performed to control the protein loading. bLF in the whole cell lysate was detected by western blotting, and immunoglobulin G (IgG) antibody was used as a negative control.
(TIF)

S3 Fig (A) Synovial fibroblast cells from rheumatoid arthritis patients (RASF) were pretreated with 0, 1, 10, and 100 μg/mL bovine lactoferrin (bLF) for 4 h before tumor necrosis factor α (TNF-α) (10 ng/mL) stimulation. The interleukin 17 (IL-17) concentration in the culture medium was measured using ELISA. (B) IL-17 secretion by spleen cells isolated from the arthritis (Ar), Ar+liposomal bLF (LbLF) 100, and Ar+LbLF500 groups was analyzed by ELISA. *, p<0.05; **, p<0.01.
(TIF)

S1 Raw images (PPTX)
