# Supplementary figures and images for: Oral administration of bovine lactoferrin suppresses the progression of rheumatoid arthritis in an SKG mouse model
Source: PLoS One. 2022 Feb 11;17(2):e0263254. doi: 10.1371/journal.pone.0263254 (PMC8836292; doi:10.1371/journal.pone.0263254)

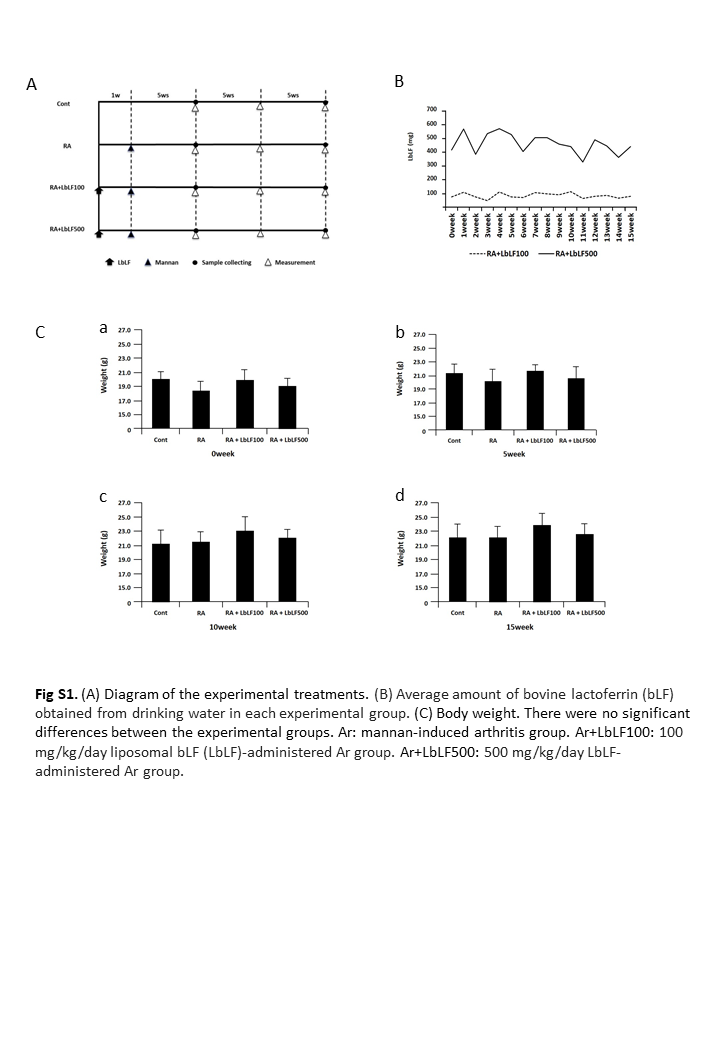

Supplement: S1 Fig — (A) Diagram of the experimental treatments. (B) Average amount of bovine lactoferrin (bLF) obtained from drinking water in each experimental group. (C) Body weight. There were no significant differences between the experimental groups. Ar: Mannan-induced arthritis group. Ar+LbLF100: 100 mg/kg/day liposomal bLF (LbLF)-administered Ar group. Ar+LbLF500: 500 mg/kg/day LbLF-administered Ar group. (TIF) [file pone.0263254.s001.tif]

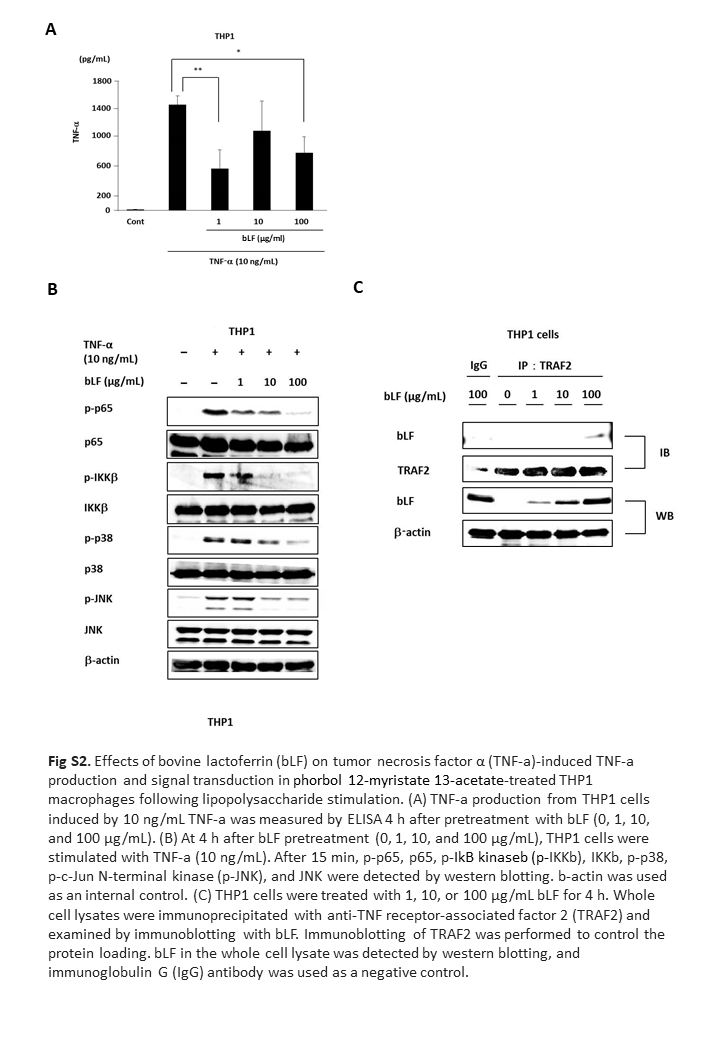

Supplement: S2 Fig — (A) TNF-α production from THP1 cells induced by 10 ng/mL TNF-α was measured by ELISA 4 h after pretreatment with bLF (0, 1, 10, and 100 μg/mL). (B) At 4 h after bLF pretreatment (0, 1, 10, and 100 μg/mL), THP1 cells were stimulated with TNF-α (10 ng/mL). After 15 min, p-p65, p65, p-IκB kinase β (p-IKKβ), IKKβ, p-p38, p-c-Jun N-terminal kinase (p-JNK), and JNK were detected by western blotting. β-actin was used as an internal control. (C) THP1 cells were treated with 1, 10, or 100 μg/mL bLF for 4 h. Whole cell lysates were immunoprecipitated with anti-TNF receptor-associated factor 2 (TRAF2) and examined by immunoblotting with bLF. Immunoblotting of TRAF2 was performed to control the protein loading. bLF in the whole cell lysate was detected by western blotting, and immunoglobulin G (IgG) antibody was used as a negative control. (TIF) [file pone.0263254.s002.tif]

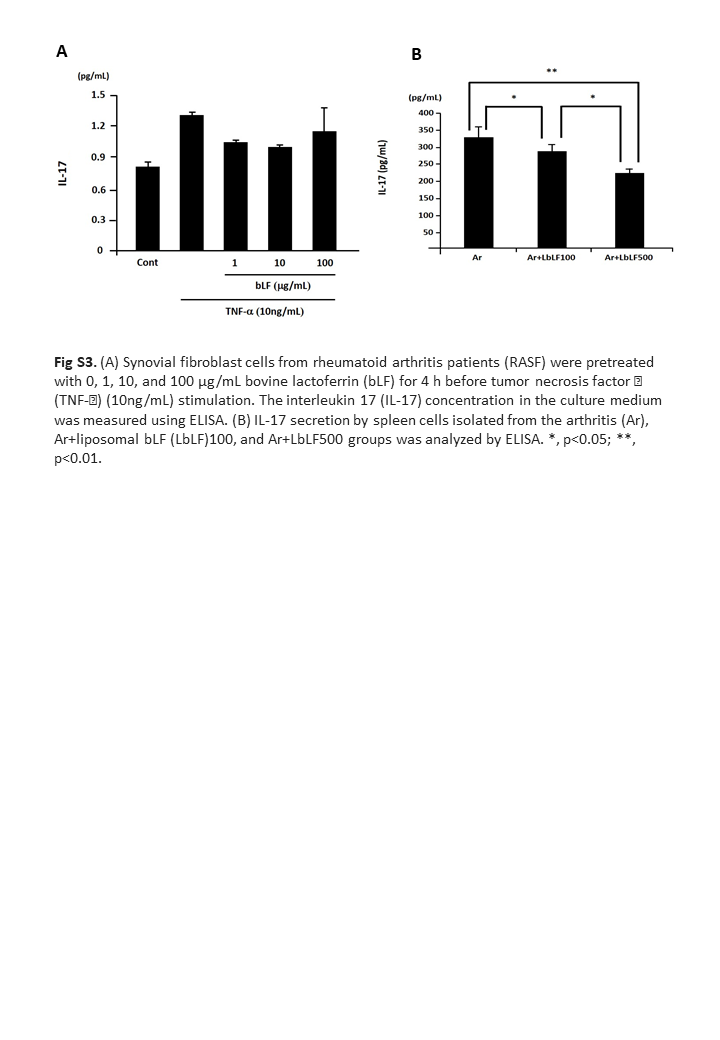

Supplement: S3 Fig — (A) Synovial fibroblast cells from rheumatoid arthritis patients (RASF) were pretreated with 0, 1, 10, and 100 μg/mL bovine lactoferrin (bLF) for 4 h before tumor necrosis factor α (TNF-α) (10 ng/mL) stimulation. The interleukin 17 (IL-17) concentration in the culture medium was measured using ELISA. (B) IL-17 secretion by spleen cells isolated from the arthritis (Ar), Ar+liposomal bLF (LbLF) 100, and Ar+LbLF500 groups was analyzed by ELISA. *, p<0.05; **, p<0.01. (TIF) [file pone.0263254.s003.tif]
