# Supplementary material for: Oral administration of bovine lactoferrin suppresses the progression of rheumatoid arthritis in an SKG mouse model
Source: PLoS One. 2022 Feb 11;17(2):e0263254. doi: 10.1371/journal.pone.0263254 (PMC8836292; doi:10.1371/journal.pone.0263254)
Supplement: S1 Raw images — (PPTX) [file pone.0263254.s004.pptx]

## Slide 1
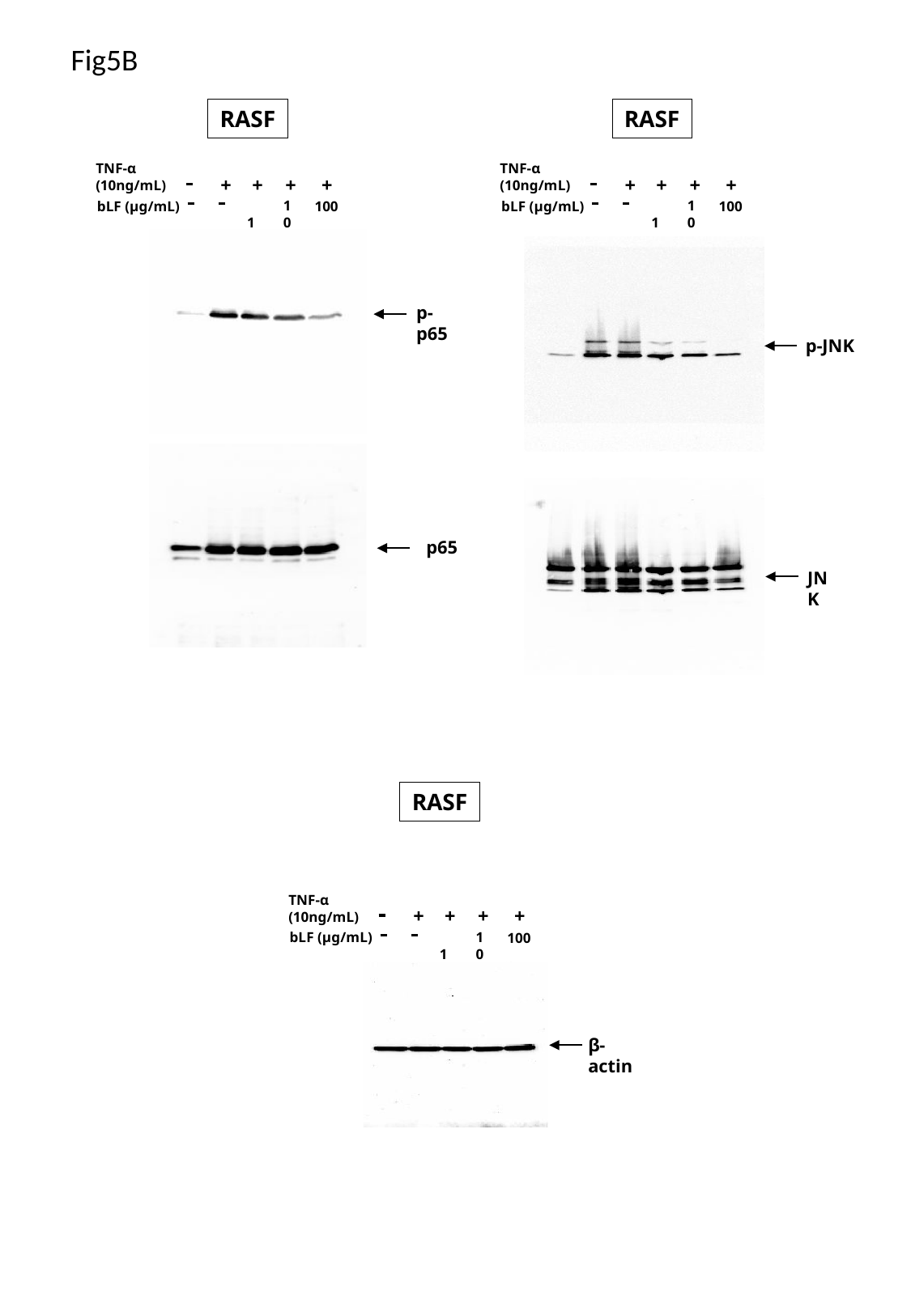

Fig5B
RASF
RASF
TNF-α
(10ng/mL)
TNF-α
(10ng/mL)
+
+
+
+
+
+
+
+
10
10
 1
 1
bLF (μg/mL)
bLF (μg/mL)
100
100
p-p65
p-JNK
p65
JNK
RASF
TNF-α
(10ng/mL)
+
+
+
+
10
 1
bLF (μg/mL)
100
β-actin

## Slide 2
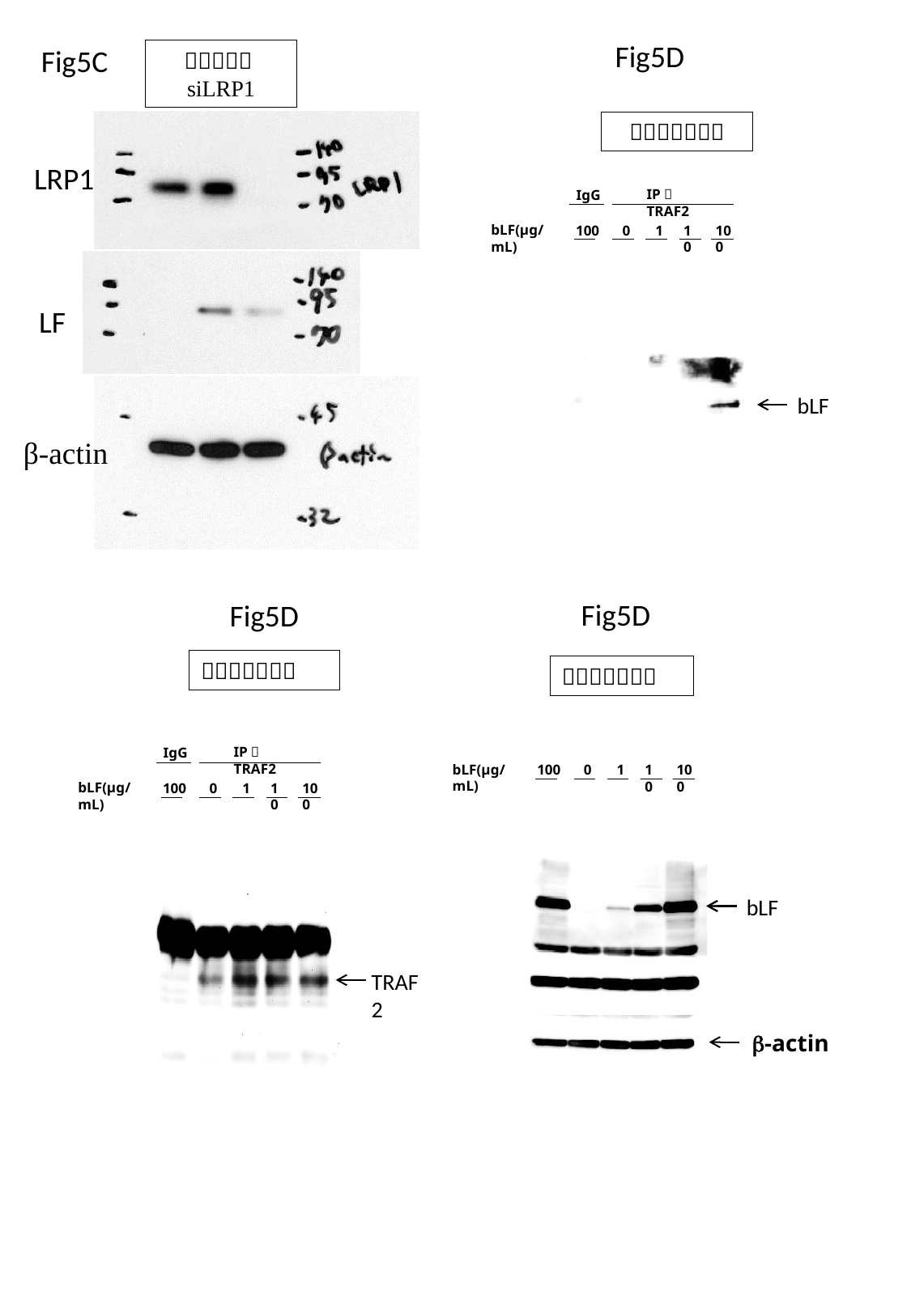

Fig5D
Fig5C
ＲＡＳＦ：siLRP1
ＲＡＳＦ：ＩＰ
LRP1
IP：TRAF2
IgG
bLF(μg/mL)
100
0
1
10
100
LF
bLF
β-actin
Fig5D
Fig5D
ＲＡＳＦ：ＩＰ
ＲＡＳＦ：Ｗｂ
IP：TRAF2
IgG
bLF(μg/mL)
100
0
1
10
100
bLF(μg/mL)
100
0
1
10
100
bLF
TRAF2
b-actin
